# Promoting Antibiotic Stewardship and Implementation of Sepsis Pathway in the Emergency Department: A Quality Improvement Initiative

**DOI:** 10.7759/cureus.49275

**Published:** 2023-11-23

**Authors:** Iqra Zia, Syeda Kisa Fatima Zaidi

**Affiliations:** 1 Acute Medicine, Royal Free Hospital, London, GBR; 2 Applied Clinical Research, McMaster University, Hamilton, CAN

**Keywords:** antimicrobial stewardship, care bundle, antibiotics, emergency department, sepsis

## Abstract

Introduction

Sepsis is a preventable cause of mortality and presents challenges in triage and management. The Surviving Sepsis Campaign care bundles improve patient outcomes; however, non-compliance with guidelines, understaffing, and scarcity of training opportunities undermine care quality in resource-limited countries. We aimed to implement the sepsis hour-1 care bundle in the emergency department of a tertiary-care hospital in Pakistan and develop hospital antimicrobial guidelines.

Methods

The baseline assessment included a survey of knowledge and confidence in sepsis management and a retrospective audit of inpatient medical records. The inclusion criteria were age ≥ 18 years with a systemic inflammatory response score ≥ 2 or a National Early Warning Score ≥ 3. Improvement strategies included (a) educational intervention, (b) adult sepsis screening tool and sepsis 1-hour bundle checklist, and (c) recommendations for empirical antibiotics. These were implemented and assessed via Plan-Do-Study-Act (PDSA) cycles: (a) multi-tiered educational campaigns, (b) implementation of hospital protocols/guidelines, and (c) antimicrobial policy and sustainability. The process measures were hour-1 bundle components and the outcome measures were in-hospital mortality, ICU admission, length of hospital stay, and ICU stay.

Results

The baseline survey revealed that the majority of participants had formal training and felt confident in managing septic patients but none of the respondents had used a sepsis scoring system, and only 29.4% had used an hour-1 bundle previously. There was a sustained improvement in bundle compliance from 0% at baseline to 57.7% at PDSA-3. Inappreciable variation (p > 0.05) was reflected in the length of hospital and ICU stay and in-hospital mortality, whereas ICU admission decreased insignificantly (p > 0.05). The antimicrobial therapy practice, as per the guidelines, increased remarkably (p < 0.05).

Conclusion

Regular training and feedback are pivotal for practice change, yet integrating structured screening tools and bundled checklists into current workflows can significantly improve compliance.

## Introduction

Sepsis is a life-threatening organ dysfunction due to a dysregulated body response to infection, whereby organ dysfunction is identified as an acute change in the Sequential Organ Failure Assessment (SOFA) score [1]. The mortality exceeds 40% if a patient develops septic shock defined by the presence of circulatory and metabolic/cellular abnormalities and is characterized by persistent hypotension requiring vasopressors to maintain a mean arterial pressure (MAP) of 65 mmHg and a serum lactate level of >2 mmol/L despite adequate volume resuscitation [1]. The WHO Global Report on Disease Burden and Mortality in the year 2017 revealed worldwide 48.9 million cases of sepsis and 11 million sepsis-related deaths, with the majority attributed to diarrhea, followed by lower respiratory infections [2]. It is an integral component of Sustainable Development Goals as a cause of preventable mortality, with significantly higher (approximately 85%) incidence and mortality in newborns, pregnant women, and people from lower-middle-income countries [2]. Preventive measures include universal health coverage, reduction of antimicrobial resistance, improving sanitation, hand hygiene standards, water quality, and other infection prevention control measures [2].

The Surviving Sepsis Campaign (SSC) has consistently provided evidence-based guidelines for the management of sepsis and septic shock as medical emergencies. The 2018 "hour-1" care bundle was developed with the intent of immediate resuscitation and management [3]. A care bundle offers a structured way to exercise evidence-based practice and has been proven to improve patient outcomes [4]. Successful completion of this bundle is associated with reduced mortality [5], whereas each hour of delay in antimicrobial administration for patients with septic shock is associated with a decrease in survival by 7.6% [6]. The bundle elements [3] include blood lactate level, repeating it if initial value > 2 mmol/L, blood cultures before administration of antibiotics, administration of broad-spectrum antibiotics, initiation of rapid administration of 30 ml/kg crystalloid if the patient is hypotensive or lactate ≥ 4 mmol/L, and application of vasopressors if hypotension persists during or after fluid resuscitation to maintain a MAP of ≥65 mmHg.

In Pakistan, a study revealed that 42% of mortality in a tertiary-care hospital is attributed to sepsis [7], while the most common cause was respiratory infections, followed by urinary tract infections [7-9]. Another study in the year 2017 delineated sepsis incidence of 40.9%, and septic complications were found to be the most common cause of death, i.e., 36.19% [9]. A study suggested self-reported compliance of 60% to hour-1 bundle components, and 54% of respondents indicated a lack of evidence-based protocol for antimicrobial therapy [10]. The perceived barriers to compliance in emergency settings are short staffing, delayed hospital presentation, and overcrowding [11]. A study revealed that methicillin-sensitive *Staphylococcus aureus* was the most common pathogen; however, the sensitivity pattern highlighted that the majority of the bacteria were resistant to common antibiotics, and no single antibiotic had >70% susceptibility [12]. The project aimed at early recognition of sepsis and improving compliance with the sepsis hour-1 care bundle for resuscitation and management in the emergency department (ED) of a tertiary-care hospital in Pakistan while developing hospital antimicrobial guidelines.

This article was accepted for poster presentation at Infection Prevention Conference 2023 in Liverpool on 19th October 2023.

## Materials and methods

This service improvement study was conducted at the ED of a 354-bed tertiary-care hospital in Lahore, Pakistan, from June 2022 to February 2023. A multidisciplinary team was developed, and the standard of care for patients with sepsis and septic shock was defined as the Surviving Sepsis Campaign hour-1 care bundle. Ethical approval was obtained from the Institutional Review Board of the hospital (approval number: HLH/ADM/IRB/2022-012). To evaluate the effectiveness of the interventions, the process measures were defined as hour-1 bundle components, and the outcome measures were in-hospital mortality, ICU admission, length of hospital stay, and ICU stay. Recommendations by the Medical Microbiology & Infectious Diseases Society of Pakistan [13] were used as a standard to assess the appropriateness of antimicrobial prescriptions. The baseline assessment involved a survey completed by junior doctors in the ED to gauge their knowledge of and confidence in managing sepsis. Concurrently, a retrospective audit (April to March 2022) of the medical records of patients admitted to the medical inpatient ward (60 beds) meeting the inclusion criteria was conducted. The inclusion criteria were age ≥ 18 years, presenting to the ED with a systemic inflammatory response syndrome (SIRS) score ≥ 2 or a National Early Warning Score (NEWS) ≥ 3. The exclusion criteria were age < 18 years and direct admission to the ward/ICU bypassing the ED. Following baseline data collection and analysis, the findings were presented to the ward manager and medical and nursing staff. This was followed by discussion sessions among team members, the ward manager, and the head nurse to identify and devise improvement strategies after feasibility analysis. Improvement strategies included (i) educational intervention, (ii) an adult sepsis screening tool and sepsis 1-hour bundle checklist as a standard operating procedure, and (iii) recommendations for empirical antibiotics to guide appropriate therapy. Data were entered into the Statistical Package for Social Sciences (SPSS Statistics for Windows version 26, IBM Corp., Armonk, NY) for analysis. Descriptive statistics are frequency, percentage, and median with interquartile range (IQR). For normality, the Kolmogorov-Smirnov test identified a non-uniform distribution of quantitative data (p < 0.05); hence, non-parametric analysis was used. To analyze improvement in practice, the outcome measures were compared with the baseline, ICU, and hospital stay, using the Kruskal-Wallis test. ICU admission and in-hospital mortality were compared using Pearson’s chi-squared test. Statistical significance was set at p < 0.05.

The interventions were implemented and assessed via three sequential Plan-Do-Study-Act (PDSA) cycles.

PDSA cycle 1 (June to July 2022): multi-tiered educational campaign

It involved biweekly focused educational sessions targeting assessment and recognition scoring systems (SIRS and NEWS), sepsis hour-1 care bundle components, and guidelines for appropriate empirical antibiotic therapy for suspected sites and sources of infection. In addition, comprehensive case-based discussions and feedback were initiated to identify obstacles to compliance and difficulties junior doctors face while managing septic patients. A senior review of all patients with severe sepsis and a critical care review of all patients with septic shock are recommended.

PDSA cycle 2 (August to October 2022): development and implementation of hospital protocols and guidelines

The lack of a structured framework and envisaging care bundles during busy ED shifts has been identified as an impediment to process improvements. However, doctors rarely missed identifying patients with sepsis, regardless of whether compliance was suboptimal. Given that, a sepsis screening tool/clinical pathway and hour-1 care bundle for ED were developed as a goal of care, fostering a robust triage and management system. The care pathway was adapted from the Oxford Academic Health Safety Network [14] and was endorsed and reviewed by team members.

PDSA cycle 3 (November 2022 to January 2023): antimicrobial policy and sustainability

While regular feedback and reminders continued to improve the standards of sepsis care, education related to the pathway was incorporated into orientation sessions for junior doctors. To optimize antimicrobial prescription practices, hospital guidelines were developed as "Recommendations for Empirical Antibiotics."

## Results

A total of 176 patients were reviewed during the study, and there were no conflicts between data collaborators assessing the patients' eligibility.

Baseline assessment

**Figure 1:**
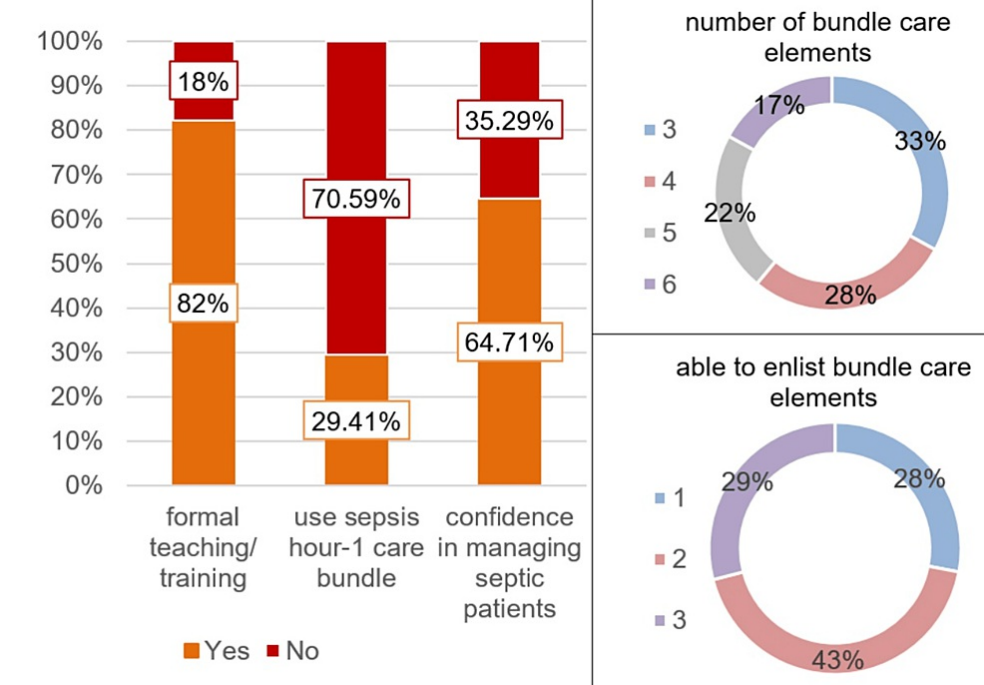
Junior doctors’ survey of knowledge of sepsis and confidence in management

The survey form was distributed to 27 junior doctors and was filled by 17 (response rate = 62.96%). Among the respondents, 10 (58.82%) were medical officers and seven (41.18%) were internal medicine residents. While the majority had attended a formal teaching/training (82%) and were confident in managing septic patients (64.71%), none of them used any scoring system for recognition of septic patients, and only 24.91% had used sepsis hour-1 care bundle previously (Figure 1). Regarding knowledge of the sepsis hour-1 bundle (Figure 1), only 22% identified the number of elements, and the majority (43%) enlisted only two interventions. Furthermore, an audit of medical records suggested compliance of 0% with the hour-1 bundle and 26.70% of antimicrobial prescriptions as per recommended guidelines.

Process measures

**Figure 2:**
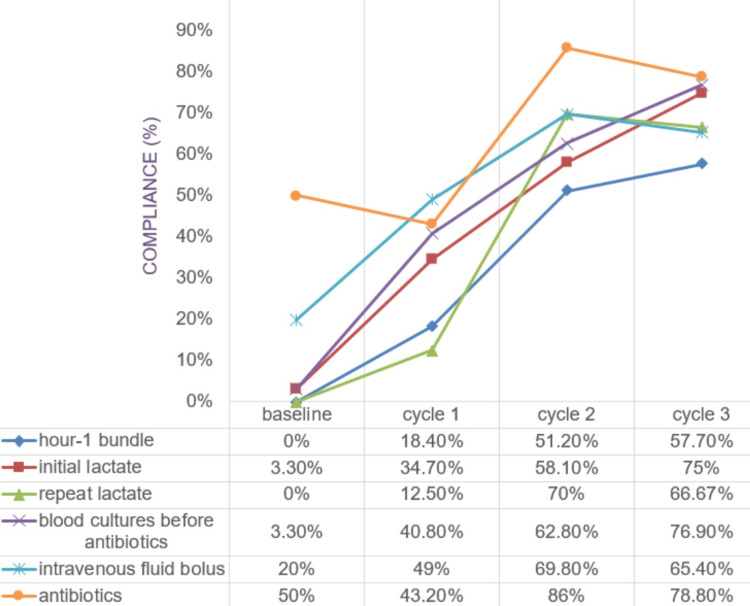
Compliance of sepsis care bundle

There was a sustained improvement in the compliance of the hour-1 bundle across all cycles in the post-intervention phase (Figure 2). Notably, an increase in compliance was observed after the introduction of the sepsis care pathway, from 18.4% to 51.2%. Among the components, a remarkable increase in compliance was observed for initial lactate (3.3% to 75%), repeat lactate if required (0% to 66.67%), blood cultures before antibiotics (3.3% to 76.9%), and intravenous fluid bolus (20% to 65.40%). Interestingly, while 50% of cases received antibiotics administered on time at baseline, there was a sharp decrease from cycle 2 to cycle 3 (from 86% to 78.80%).

The patients with "severe sepsis" being reviewed by a senior doctor increased from 46.7% at baseline to 53.1% at cycle 1, 79.1% at cycle 2, and 92.3% at cycle 3. The review of "septic shock" patients by the critical care team also increased from 23.3% at baseline to 20.4% at cycle 1, 39.5% at cycle 2, and 65.4% at cycle 3.

Outcome measures

**Figure 3:**
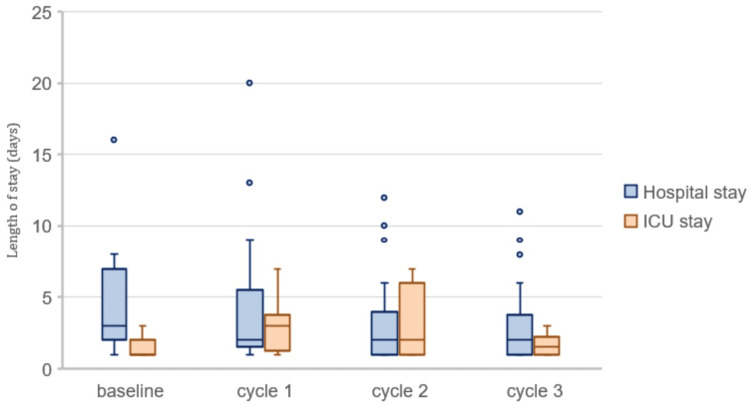
Impact of intervention on the length of hospital and ICU stay

From the baseline assessment to cycle 3 (Figure 3), an insignificant decrease (p = 0.101) in the duration of hospital stay from a median value of 3 (IQR = 2-7) to 2 (IQR = 1-3.75) can be appreciated. With regards to the length of ICU stay (Figure 3), there was an insignificant increase (p = 0.317) from 1 (IQR = 1-2) at baseline to 1.5 (IQR = 1-2.25) at cycle 3.

**Figure 4:**
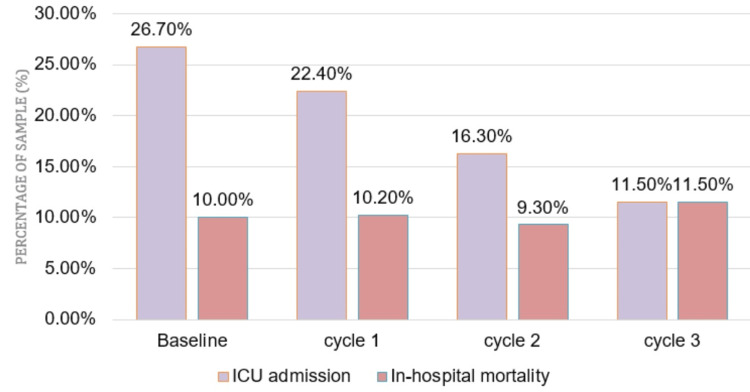
Impact of intervention on ICU admission and in-hospital mortality

There was a continuing decrease in ICU admission (Figure 4) from 26.7% to 11.5% at baseline and cycle 3, respectively; however, the variation was reported as insignificant (p = 0.257). For in-hospital mortality (Figure 4), a minor increasing trend was observed from 10% (baseline) to 11.5% (cycle 3), which was statistically insignificant (p = 0.051).

**Figure 5:**
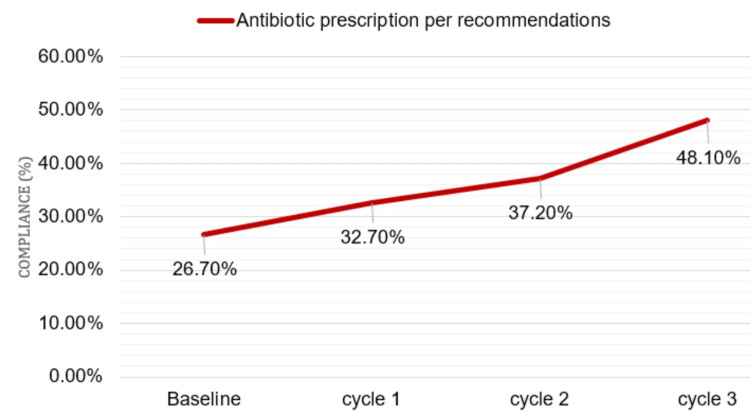
Impact of intervention on antimicrobial therapy

Regarding antimicrobial therapy compliance with the recommended guidelines (Figure 5), a significant improvement (p = 0.013) was identified, from 26.70% at baseline to 48.10% at cycle 3.

## Discussion

Sepsis is frequently misdiagnosed in the ED, underscoring the urgent need for early detection. Our study focused on the timely identification of sepsis using the "Adult Sepsis Screening Tool" and implementation of the sepsis hour-1 care bundle, complemented by the development of hospital antimicrobial guidelines. Initially, the healthcare workers reported having formal training (82%) and confidence in managing septic patients (64.71%); however, they exhibited sub-optimal knowledge of sepsis scoring systems and hour-1 bundles (Figure 1), which was reflected by hour-1 care bundle compliance of 0%. The finding is similar to another study that revealed that the knowledge of 85% of the participants regarding SSC guidelines was “very familiar” or “somewhat familiar,” and 84% of the participants reported their training in the diagnosis and management of sepsis as “excellent” or “good"; however, only <20% of the study population correctly identified sepsis diagnostic criteria [15]. Through a comprehensive approach involving educational interventions, and the introduction of screening tools and checklists, substantial enhancements in bundle compliance were achieved, increasing from an initial 0% to a commendable 57.7% at the end of cycle 3 (Figure 2). While there were minor fluctuations in certain outcome measures (Figures 3, 4), such as length of hospital and ICU stay, ICU admission, and in-hospital mortality, these changes were statistically insignificant. The successful implementation of these pathways benefits healthcare systems in terms of cost-effectiveness and resource optimization by reducing unnecessary testing, lowering admission rates, and shortening hospital stays through standardized care [16].

The implications of the findings of this study are substantial. They suggested that healthcare institutions should prioritize ongoing education for physicians to enhance sepsis recognition and management skills. The study showed that care bundle compliance led to an insignificant increase in in-hospital mortality (from 10% to 11.5%). This finding is similar to another study that delineated a 7% decrease in mortality following bundle adherence among ICU patients while no significant improvement was signified in the ward and ED [17]. Similarly, a multi-center study in Brazil found that the time to sepsis diagnosis was independently associated with a reduction in mortality risk, whereas six-hour bundle compliance was not associated [18]. It is noteworthy that similar studies with comparable patient characteristics at baseline and post-intervention showed significant improvements in process measures, including adherence to all elements of the three-hour sepsis bundle, timely antibiotic administration in the one-hour septic shock bundle, and a substantial decrease in ICU admission [19].

The study has some limitations. The study was conducted in a single setting, which may limit the generalizability of the findings. Our study did not address certain confounding factors, such as demographics, co-morbidities sepsis severity, and organ dysfunction, which could have affected the outcomes. Finally, the lack of follow-up post-discharge limits the assessment of long-term effects.

It is important to note that the extensive use of antibiotics coupled with elevated resistance rates necessitates the development of a policy to encourage prudent usage. To enhance the appropriateness of empirical antibiotic therapy for sepsis patients, it is valuable to consider local infection site patterns and the results of antibiotic sensitivity tests as supporting data [20]. A study implementing a three-tier approach comprising a nurse-driven screening tool, a computer-assisted screening algorithm that generated a sepsis alert in the electronic medical records, and automated sepsis-specific order sets for initial workup, resuscitation, antibiotic selection, and goal-directed therapy has shown promising results in expediting care delivery to sepsis patients in the ED [21]. Furthermore, early recognition of sepsis using electronic health records improved compliance with sepsis bundles [22].

## Conclusions

To promote patient safety and care process, the comprehensive initiative focused on compliance with the sepsis hour-1 care bundle in ED settings. The collective implementation of these interventions has the potential to improve patient outcomes and reduce unnecessary utilization of hospital resources during prolonged hospital stays. Moreover, conducting long-term follow-up studies is crucial to assess the sustained impact of these interventions. This would not only enhance the quality of healthcare services but also alleviate the physician burden.

## Figures and Tables

**Figure 6:**
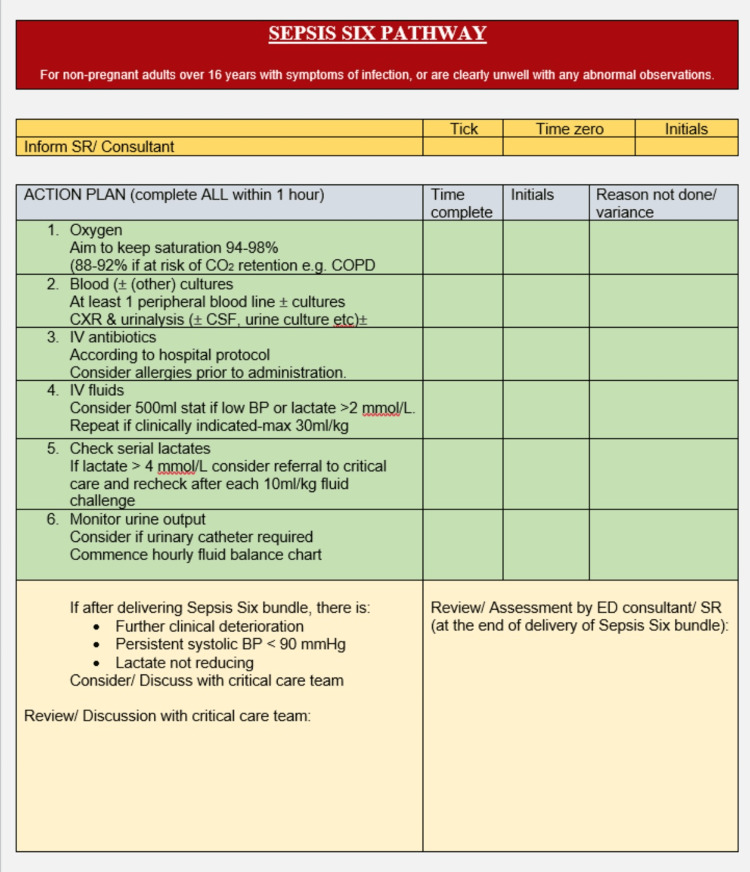
Sepsis hour-1 care bundle

**Figure 7:**
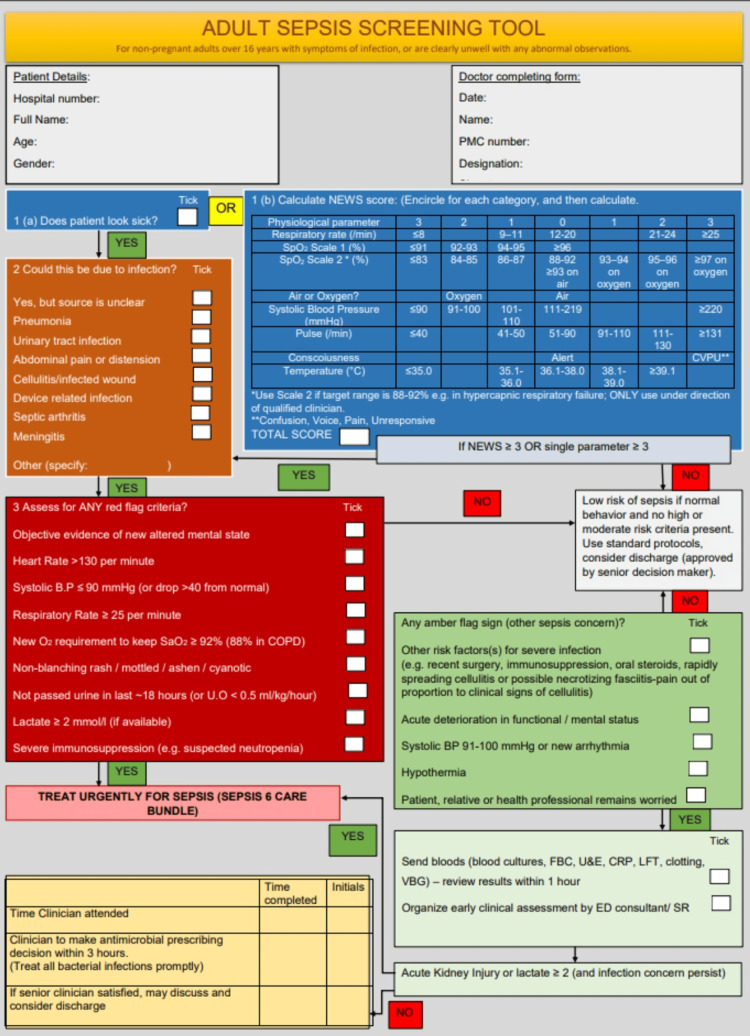
Adult sepsis screening tool

**Figure 8:**
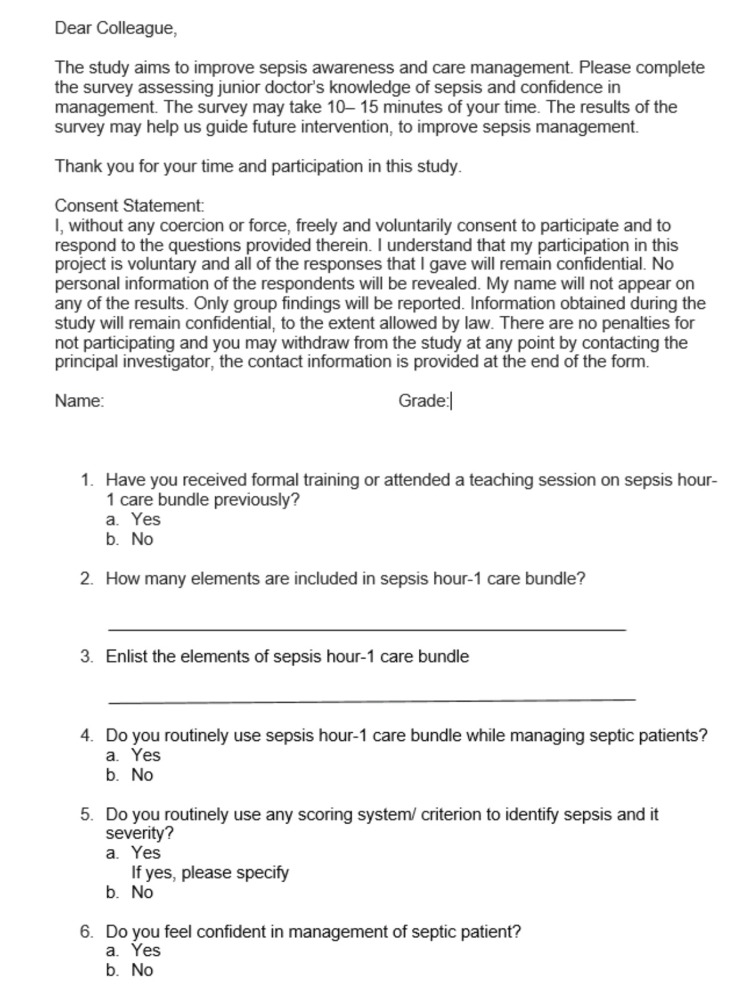
Junior doctor's survey of knowledge of sepsis and confidence in management

## References

[1] Singer M, Deutschman CS, Seymour CW (2016). The Third International Consensus Definitions for Sepsis and Septic Shock (Sepsis-3). JAMA.

[2] (2020). World Health Organization. Global report on the epidemiology and burden of sepsis: current evidence, identifying gaps and future directions. https://www.who.int/publications/i/item/9789240010789.

[3] Levy MM, Evans LE, Rhodes A (2018). The Surviving Sepsis Campaign bundle: 2018 update. Intensive Care Med.

[4] (2022). What is a bundle?. http://www.ihi.org/resources/Pages/ImprovementStories/WhatIsaBundle.aspx.

[5] Daniels R, Nutbeam T, McNamara G, Galvin C (2011). The sepsis six and the severe sepsis resuscitation bundle: a prospective observational cohort study. Emerg Med J.

[6] Kumar A, Roberts D, Wood KE (2006). Duration of hypotension before initiation of effective antimicrobial therapy is the critical determinant of survival in human septic shock. Crit Care Med.

[7] Arshad A, Ayaz A, Haroon MA, Jamil B, Hussain E (2020). Frequency and cause of readmissions in sepsis patients presenting to a tertiary care hospital in a low middle income country. Crit Care Explor.

[8] Nasir N, Jamil B, Siddiqui S, Talat N, Khan FA, Hussain R (2015). Mortality in sepsis and its relationship with gender. Pak J Med Sci.

[9] Azim S, Zahoor S, Janjua J, Majeed A, Hussain SW (2017). Analysis of pattern of mortality in medicine and allied departments at a tertiary care hospital in Islamabad: a losing battle against sepsis. J Pak Med Assoc.

[10] Feroze R, Tariq M, Toor AW (2021). Compliance to surviving sepsis campaign hour-1 bundle-a cross sectional study among physicians involved in critical care in Pakistan. Pak Armed Forces Med J.

[11] Ismail M, Aftab U, Azizi K, Khan BA (2021). Knowledge, attitudes, practices and perceived barriers of emergency health care providers regarding sepsis and septic shock in a tertiary care centre: a cross-sectional study. J Pak Med Assoc.

[12] Rehman ZU, Hassan Shah M, Afridi MNS, Sardar H, Shiraz A (2021). Bacterial sepsis pathogens and resistance patterns in a South Asian tertiary care hospital. Cureus.

[13] Hashmi M, Khan FH, Zubairi S (2015). Developing local guidelines for management of sepsis in adults: sepsis guidelines for Pakistan (SGP). Anaesth Pain Intensive Care.

[14] Brent Brent, A. (2017, January 5 (2017). From confusion to consensus: the Oxford AHSN sepsis pathway. https://www.patientsafetyoxford.org/wp-content/uploads/2017/05/Andrew-Brent-Oxford-AHSN-Regional-Sepsis-Pathway-PSC-National-Event-May-2017.pdf.

[15] Watkins RR, Haller N, Wayde M, Armitage KB (2020). A multicenter survey of house staff knowledge about sepsis and the “Surviving Sepsis Campaign guidelines for management of severe sepsis and septic shock”. J Intensive Care Med.

[16] Lavelle J, Schast A, Keren R (2015). Standardizing care processes and improving quality using pathways and continuous quality improvement. Curr Treat Options Peds.

[17] Milano PK, Desai SA, Eiting EA, Hofmann EF, Lam CN, Menchine M (2018). Sepsis bundle adherence is associated with improved survival in severe sepsis or septic shock. West J Emerg Med.

[18] Machado FR, Ferreira EM, Schippers P (2017). Implementation of sepsis bundles in public hospitals in Brazil: a prospective study with heterogeneous results. Crit Care.

[19] Venkatesh B, Schlapbach L, Mason D (2022). Impact of 1-hour and 3-hour sepsis time bundles on patient outcomes and antimicrobial use: a before and after cohort study. Lancet Reg Health West Pac.

[20] Pradipta IS, Sodik DC, Lestari K, Parwati I, Halimah E, Diantini A, Abdulah R (2013). Antibiotic resistance in sepsis patients: evaluation and recommendation of antibiotic use. N Am J Med Sci.

[21] Warstadt NM, Caldwell JR, Tang N, Mandola S, Jamin C, Dahn C (2022). Quality initiative to improve emergency department sepsis bundle compliance through utilisation of an electronic health record tool. BMJ Open Qual.

[22] Gatewood MO, Wemple M, Greco S, Kritek PA, Durvasula R (2015). A quality improvement project to improve early sepsis care in the emergency department. BMJ Qual Saf.

